# Sustained Downregulation of Vascular Smooth Muscle Acta2 After Transient Angiotensin II Infusion: A New Model of “Vascular Memory”

**DOI:** 10.3389/fcvm.2022.854361

**Published:** 2022-03-14

**Authors:** Lucie Pothen, Roxane Verdoy, Delphine De Mulder, Hrag Esfahani, Charlotte Farah, Lauriane Y. M. Michel, Flavia Dei Zotti, Bertrand Bearzatto, Jerome Ambroise, Caroline Bouzin, Chantal Dessy, Jean-Luc Balligand

**Affiliations:** ^1^Institute of Experimental and Clinical Research (IREC), Pole of Pharmacology and Therapeutics (FATH), Cliniques Universitaires St-Luc and Université Catholique de Louvain (UCLouvain), Brussels, Belgium; ^2^Institute of Experimental and Clinical Research (IREC), Centre des Technologies Moléculaires Appliquées (CTMA), Cliniques Universitaires St-Luc and Université Catholique de Louvain (UCLouvain), Brussels, Belgium; ^3^Institute of Experimental and Clinical Research (IREC), Imaging Platform (2IP), Cliniques Universitaires St-Luc and Université Catholique de Louvain (UCLouvain), Brussels, Belgium

**Keywords:** angiotensin II, aortic tissue, VSMC, memory, ACTA2, smooth muscle actin (SMA)

## Abstract

**Background:**

Activation of the renin-angiotensin-aldosterone system (RAAS) plays a critical role in the development of hypertension. Published evidence on a putative “memory effect” of AngII on the vascular components is however scarce.

**Aim:**

To evaluate the long-term effects of transient exposure to AngII on the mouse heart and the arterial tissue.

**Methods:**

Blood pressure, cardiovascular tissue damage and remodeling, and systemic oxidative stress were evaluated in C57/B6/J mice at the end of a 2-week AngII infusion (*AngII*); 2 and 3 weeks after the interruption of a 2-week AngII treatment (*AngII*+*2W* and *AngII* +*3W*; so-called “memory” conditions) and control littermate (*CTRL*). RNAseq profiling of aortic tissues was used to identify potential key regulated genes accounting for legacy effects on the vascular phenotype. RNAseq results were validated by RT-qPCR and immunohistochemistry in a reproduction cohort of mice. Key findings were reproduced in a homotypic cell culture model.

**Results:**

The 2 weeks AngII infusion induced cardiac hypertrophy and aortic damage that persisted beyond AngII interruption and despite blood pressure normalization, with a sustained vascular expression of ICAM1, infiltration by CD45+ cells, and cell proliferation associated with systemic oxidative stress. RNAseq profiling in aortic tissue identified robust *Acta2* downregulation at transcript and protein levels (α-smooth muscle actin) that was maintained beyond interruption of AngII treatment. Among regulators of *Acta2* expression, the transcription factor Myocardin (*Myocd*), exhibited a similar expression pattern. The sustained downregulation of *Acta2* and *Myocd* was associated with an increase in H3K27me3 in nuclei of aortic sections from mice in the “memory” conditions. A sustained downregulation of *ACTA2* and *MYOCD* was reproduced in the cultured human aortic vascular smooth muscle cells upon transient exposure to Ang II.

**Conclusion:**

A transient exposure to Ang II produces prolonged vascular remodeling with robust *ACTA2* downregulation, associated with epigenetic imprinting supporting a “memory” effect despite stimulus withdrawal.

## Introduction

Hypertension is a well recognized cardiovascular risk factor, causing up to 7.6 million deaths per year worldwide (13.5% of total deaths) (1). Usually, it develops as a slow and gradual increase in blood pressure, with occasional acute hypertensive peaks. This silent disease can remain unrecognized until potentially fatal complications occur, such as hypertrophic cardiomyopathy, strokes, or ischemic heart disease.

Hypertension is conventionally associated with a neurohormonal activation from the sympathetic nervous and the renin-angiotensin-aldosterone systems (RAAS) (2, 3). The RAAS is involved in numerous physiological functions, including vasoconstriction, fluid volume regulation, cardiac remodeling, cell growth, and vascular wall integrity. Angiotensin II (AngII), as the main product and effector of the RAAS, is a potent regulator of blood pressure (4, 5). As such, it is also a key player in hypertension development, mainly through activation of the type 1 AngII receptors (AT_1_R), that triggers structural remodeling and inflammation in the heart and vascular tissue (6). In particular, cellular processes underlying vascular injury include, among others, impaired endothelial function and a phenotypic switch of vascular smooth muscle cells (VSMCs), characterized by a reduced expression of myofibrillar proteins and contractility, evolving toward a more proliferative and synthetic state, with increased production, e.g., of proteoglycans (7–9).

Usually, activation of the RAAS accompanies the development of other cardiovascular risk factors than hypertension, such as diabetes or metabolic syndrome, including hypercholesterolemia. The resulting cardiovascular pathologies evolve with sustained deleterious effects despite the removal of the pathogenic stimulus (e.g., hyperglycemia), a phenomenon that has been coined “metabolic memory” (10). In the previous models of diabetes, temporary exposure to hyperglycemia leads to an epigenetic imprinting in endothelial cells, with sustained mitochondrial oxidative stress despite glycemic normalization (11, 12). Similar epigenetic mechanisms might explain the enduring increase in cardiovascular risk, despite glycemic control in the clinical studies, named as legacy effect (13, 14). The specific participation of the RAAS, particularly of AngII, in these enduring effects is less clear. Intriguingly, other clinical trials suggest that treatement with a RAAS inhibitor, e.g., sartans that inhibit AngII binding to the AT_1_R, could prevent organ damage and reduce cardiovascular events through protective effects beyond blood pressure lowering in hypertensive patients (15). Published evidence on a putative “memory effect” of AngII is scarce. One previous study observed a sustained vascular injury with persistent activation of multiple signaling pathways (JNK1/2, STAT3, and NF-κB) and increase in the reactive oxygen species (ROS) production up to 1 week after withdrawal of an initial AngII infusion in mice; the data suggested a link with persistent NADPH oxidase activation. However, the upstream mechanism for this sustained oxidative stress was not established (16, 17).

Surprisingly, while transcriptomic profiles of AngII-treated organs/tissues are available in the kidneys (18), heart (19), or abdominal aortic aneurysm in ApoE-/- mice (20), the AngII treatment protocol varies widely between studies, and transcriptomic data on the arterial tissue of WT mice were rarely reported (21–23), with no attempt to analyze any legacy effect.

In this study, we developed an AngII “memory” model in which we examined long-term effects of temporary exposure to AngII on heart and arterial tissue, including cardiovascular remodeling and VSMC phenotypic switch. Longitudinal transcriptomic profiling of aortic tissues after AngII withdrawal identified uniquely regulated genes potentially involved in legacy effects on the vascular phenotype.

## Materials and Methods

### Animal Experimental Protocol

**Figure 1:**
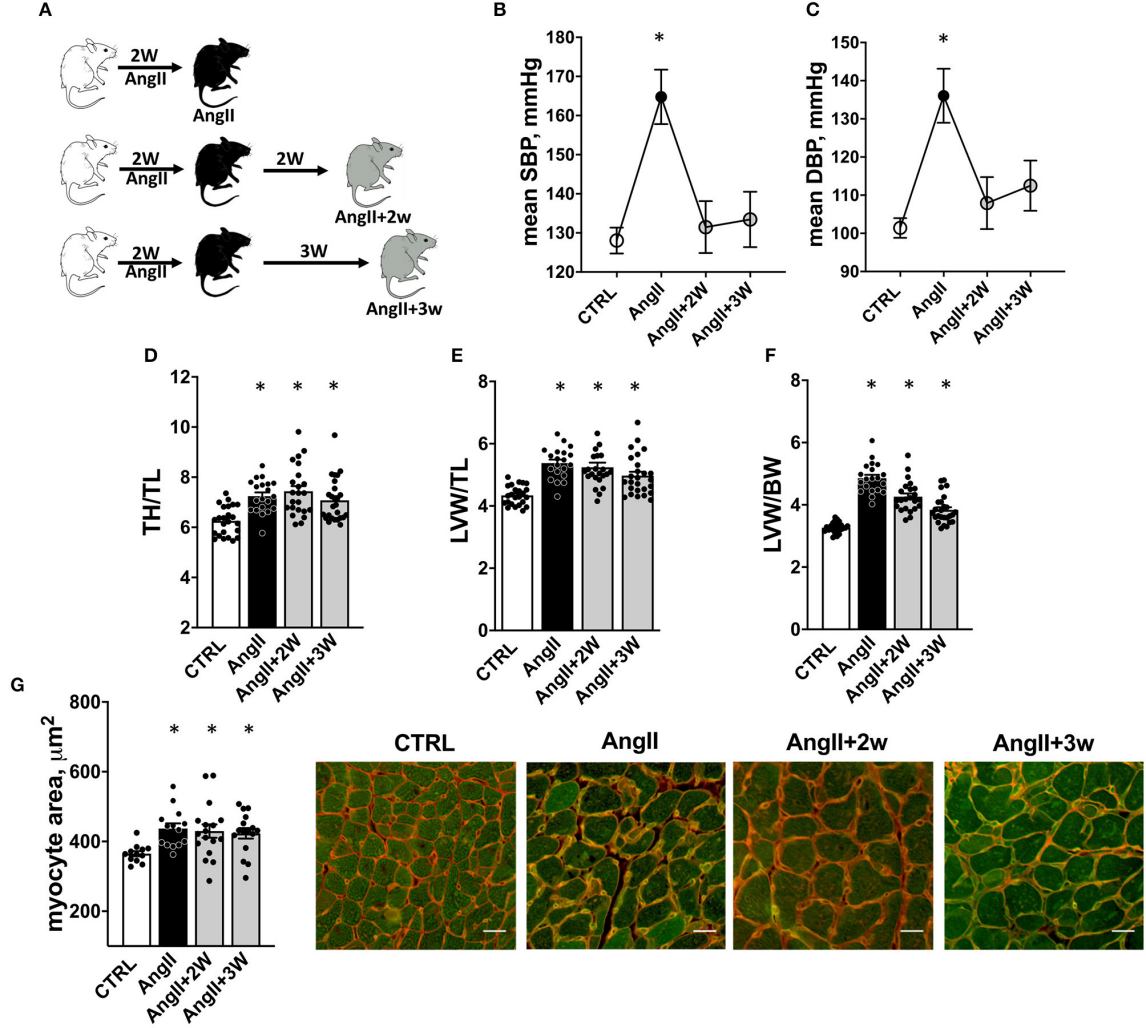
Experimental procedure and cardiac hypertrophy. Experimental procedure: **(A)** C57Bl6 mice were implanted with osmotic minipump delivering 2.8mg/kg/d of AngII during 2 weeks. One group was sacrificed at the end of the treatment (*AngII*), 2 others were sacrificed, respectively, 2 weeks (*AngII*+*2W*) and 3 weeks (*AngII*+*3W*) after the end of the minipump, and were considered as “memory groups”. Control mice (*CTRL*) were littermate age-matched mice sacrificed at each time point (2 weeks of AngII, 2 weeks of AngII + 2 weeks, 2 weeks of AngII +3 weeks). **(B)** Systolic and **(C)** Diastolic blood pressure measured with implanted telemetry devices. *n* = 19; *p < 0.05, one-way ANOVA for repeated measures, mixed-effect analysis followed by Tukey's multiple comparison test. **(D–G)** Cardiac Hypertrophy: **(D)** Total heart/tibial length ratio (TH/TL); **(E)** Left ventricular weight/tibial length ratio (LVW/TL); and **(F)** Left ventricular weight/body weight ratio (LVW/BW). *n* = 93 (initial + replication cohorts); * *p* < 0.05, one-way ANOVA followed by Holm-Sidak's multiple comparisons test. **(G**) Quantification of myocyte size area by isolectin/WGA staining and representative pictures (white bar scale = 20 μm). *n* = 59; *p < 0.05, one-way ANOVA followed by Dunett's multiple comparisons test.

11-week male C57B6/J mice were implanted with osmotic minipumps delivering of 2.8 mg/kg/d of AngII (Sigma, A9525) for 2 weeks. To confirm the effect of the pharmacological treatment, blood pressure signals from aortic arch before (*CTRL*); under AngII up to 2 weeks (*AngII*); and 2 and 3 weeks after the interruption of AngII (*AngII*+*2W*; *AngII*+*3w*, respectively) were recorded in selected, conscious, and unrestrained animals, with surgically implanted miniaturized telemetry devices (DSI, USA) as described previously (24). Mice were sacrificed after anesthesia at the same time points and age-matched littermate served as control (Figure 1A). All the investigations conformed to the Guide for the Care and Use of Laboratory Animals (NIH Publications no. 8023, revised 2011) and were approved by the Institutional Animal Care and Research Advisory Committee of the Université Catholique de Louvain.

### Histomorphometric Analysis of Hearts

Morphometric and histologic measurements were obtained from hearts arrested in diastole in KCl solution, washed, subsequently fixed with 4% formaldehyde and paraffin embedded. To assess cardiac myocyte transverse area, tissue was costained with wheat germ agglutinin (WGA; for plasma membrane staining, rhodamine-conjugated) and isolectin B4 (GS-IB4; for endothelial staining, biotin-conjugated and revealed with fluorescein-conjugated streptavidin). Cell area from 150 to 200 cells per slide was determined using AxioVision software 4.8.2.0.

### Immunostaining on Carotid Arteries and Aortas

Vessels (carotid arteries and aortas in totality) were carefully dissected, washed in cold PBS, and divided in several pieces for further experiments. One piece was subsequently fixed with 4% formaldehyde and paraffin embedded. To assess endothelial activation, carotid sections were stained with ICAM-1 primary antibody (R&D #AF796, 1/1000). On the aortic tissue, to evaluate inflammatory cells infiltration and proliferation, sections were stained with anti-CD45 primary antibody (BD Biosciences #55053P, 1/50) or rabbit anti-Ki67 primary antibody (CST #12202, 1/200), respectively. Smooth muscle actin and H3K27me3 stainings were performed using anti-αSMA primary antibody (CST #19245, 1/400) and anti-tri-methyl-histone H3 antibody (CST #9733, 1/200). Primary antibodies were revealed with EnVision-HRP systems (Agilent) and DAB (Agilent). Finally, nuclei were counterstained with H&E (Agilent). Slides were digitized with a slide scanner (SCN400 Leica) and blindly analyzed with QuPath software (University of Edinburgh) (25).

### RNA-seq on Aortic Tissue

One-third of aorta was immediately frozen in liquid nitrogen in dry Eppendorf. Because of low yield of RNA extracted in preliminary experiments, and to ensure sufficient amount and quality of RNA for sequencing, tissues from two animals were pooled in 1 ml of tri-reagent (TR118, MRC) for subsequent homogenization with the use of a Precellys Evolution homogenizer (Bertin Instrument, France). Total RNA was extracted with the PureLink™ RNA Micro Scale Kit (Invitrogen) according to the manufacturer's instructions, including a DNase step. In total, RNA from 8 samples, corresponding to 16 mice, were quantified by Qubit RNA BR assay kit (Thermo Fisher Scientific, Q10211) on a Qubit 4 Fluorometer (Thermo Fisher Scientific). RNA integrity was evaluated on the Agilent 2100 Bioanalyzer using the RNA 6000 nanokit (Agilent, 5067-1511). All the samples had RNA integrity number values between 6.8 and 7.7.

Libraries were prepared starting from 150 ng of total RNA using the KAPA RNA HyperPrep Kit with RiboErase (HMR) (KAPA Biosystems, KK8560) following the manufacturer's recommendations (KR1351—version 1.16). Libraries were equimolarly pooled and sequenced on a single lane on an Illumina NovaSeq 6000 platform. All the libraries were paired end (2 × 100 bp reads) sequenced and a minimum of 35 million paired end reads were generated per sample.

All the sequencing data were analyzed using the Automated Reproducible MOdular workflow for preprocessing and differential analysis of RNA-seq data (ARMOR) pipeline v.1.2.0 (26). In this pipeline, reads underwent a quality check using FastQC v0.11.7 (27). Quantification and quality control results were summarized in a MultiQC report (28) before being mapped using Salmon (29) to the transcriptome index which was built using all Ensembl cDNA sequences obtained in the Mus_musculus.GRCm38.cdna.all.fa (release 101) file (30). Then, the estimated transcript abundances from Salmon were imported into R using the tximeta (1.7.14) package (31, 32) and analyzed for differential gene expression with edgeR (3.31.4) package, in which *p*-value was adjusted using Benjamini-Hochberg method (33). Accordingly, each experimental group (*AngII, AngII*+*2W*, and *AngII*+*3W*) was compared against the control group (*CTRL*), thereby producing 3 lists of differentially expressed genes. Differential gene expression results from edgeR were used to conduct Over-Representation Analysis (ORA) and Gene Set Enrichment Analysis (GSEA) with the WebGestaltR (v.0.4.3) package (34). These analyses were conducted on the Kyoto Encyclopedia of Genes and Genomes (KEGG) and Reactome database. RNA-seq full data are available in the NCBI Gene Expression Omnibus (GEO) database under the study accession code GSE175588.

### RT-qPCR on Aortas in a Replication Cohort

Total RNA was extracted from one-third of the total aorta in a replication cohort of mice, with the same protocol and extraction procedure, i.e., freeze drying, further homogenization in Trizol with Precellys Evolution homogenizer, and extraction with PureLink™ RNA Micro Scale Kit. Extracted RNA was reverse-transcribed and analyzed by quantitative polymerase chain reaction (qPCR) with *GAPDH* as housekeeping gene. The primer sequences used for qPCR are presented in Table I in Supplemental Material.

### Human Aortic Vascular Smooth Muscle Cells (HVSMCs) Culture and *in-vitro* AngII Memory Model

Human aortic vascular smooth muscle cells (HAVSMC) were purchased at ScienCell Research Laboratories. Cells were grown in full Smooth Muscle Cell medium (ScienCell #1101), in T75 flask coated with polylysine (2 μg/cm^2^); medium was renewed every 24 h as cells reached 80% confluence. After serum starvation (0.1% FBS for 18 h) cells were treated with AngII 1μM for 72 h (*AngII*), or 48 h and then 24 h in control, serum-deprived medium (*MemAngII*); and compared with the cells maintained 72 h in control, serum-deprived medium (*CTRL*). Cells between passages 4 and 8 were used for experiments. Total RNA was extracted with Maxwell Kit (Promega, #AS1340). Extracted RNA was reverse-transcribed and analyzed by quantitative polymerase chain reaction (qPCR) with *GAPDH* as housekeeping gene. The primer sequences used for qPCR are presented in Table I in Supplemental Material. For protein extraction, cells were scrapped in RIPA buffer containing proteinases and phosphates inhibitors. Denatured proteins (in Laemmli buffer) were separated by SDS-PAGE and transferred on PVDF membrane. Membranes were then blocked 1 h in 5% non-fat dry milk in TBS-Tween and incubated overnight at 4°C in 1% milk Tween-TBS with primary antibodies. Antibodies were αSMA (CST #19245, 1/10000) and HSP90 (BD Biosciences #610419, 1/2500). Membranes were visualized by enhanced chemiluminescence on CL-Xposure film (Thermo Fisher Scientific).

### Statistics

Statistical tests were performed using GraphPadPrism (GraphPad Software Incorporation, San Diego, California, USA). Results are reported as mean and standard error of the mean. Statistical analysis was performed using parametric or non-parametric tests where appropriate after verifying normality of values distribution. *P* < 0.05 is considered as significant with ^*^ meaning *P* < 0.05.

## Results

The experimental design is illustrated in Figure 1A. Blood pressure was recorded longitudinally in each mouse by telemetry at four time points: at baseline (*CTRL*); at the end of the 2-week treatment with Ang II (*AngII*); and 2 and 3 weeks after the end of the Ang II infusion (*AngII*+*2W* and *AngII*+*3W*, respectively); the last two composing the “memory” groups. As expected, we observed an increase in systolic and diastolic blood pressure during AngII infusion (Figures 1B,C). Importantly, blood pressure values reverted to normal levels after the end of the 2-week treatment, confirming treatment interruption and transient exposure to Ang II, as planned. We next evaluated specific parameters of cardiovascular remodeling classically affected by AngII and their eventual persistence at longer time points.

### Cardiac Hypertrophy

As expected (4, 35), we observed a significant increase in total heart/tibial length (TH/TL), left ventricular weight/tibial length (LVW/TL), and left ventricular weight/body weight (LVW/BW) ratios in the *AngII* group (Figures 1D–F). This hypertrophic phenotype was sustained in time with ratios significantly increased in the *AngII*+*2W* and *AngII*+*3W* groups (Figures 1D–F). This was reflected by concordant increases in cardiac myocyte transverse area in the *AngII* group (Figure 1G), which remained significantly elevated in the *AngII*+*2W* and *AngII*+*3W* groups.

### Vascular Remodeling

**Figure 2:**
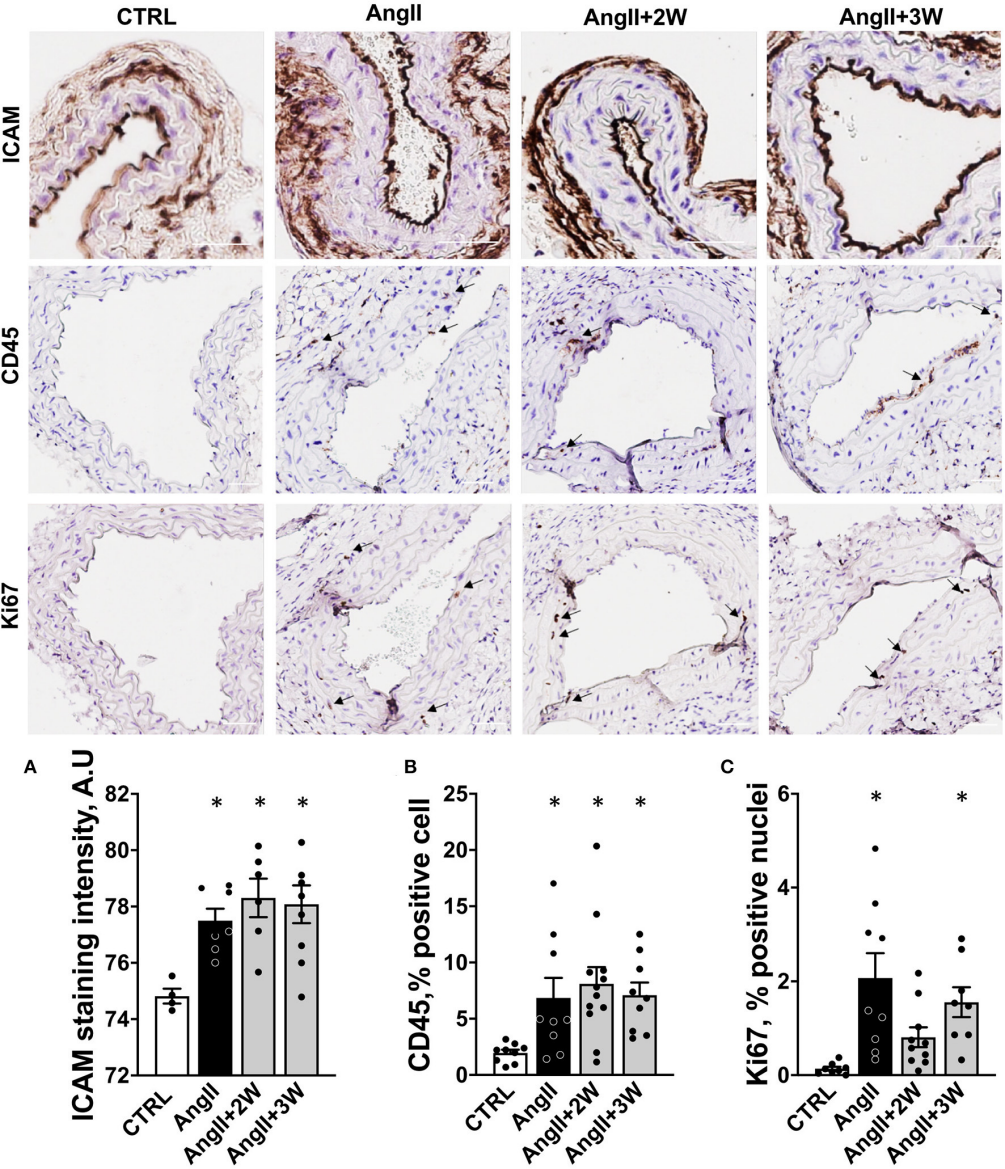
Memory effect on vascular endothelial activation, cell proliferation, and inflammation. **(A)** Quantification of endothelial staining for ICAM-1 in paraffin embedded sections of carotid arteries, expressed in arbitrary units of staining intensity, *n* = 26. **(B)** Quantification of staining for CD45 in paraffin embedded sections of aortas, expressed as % of stained cell. **(C)** Quantification of staining for Ki67 in paraffin-embedded sections of aortas, expressed as % of stained cells. *p < 0.05; *n* = 39 **(B,C)**; One-way ANOVA followed by Dunett's multiple comparisons test. Arrows indicate specific staining for CD45 and Ki67, scale bar = 100 μm.

Similar analyses were performed on the vascular phenotype. Histological analysis of carotid sections in the *AngII* group revealed an increased endothelial expression of ICAM-1, reflective of endothelial activation. ICAM-1 expression remained elevated in the *AngII*+*2W* and *AngII*+*3W* group (Figure 2A). Consistently, CD45 labeling was increased in the aortic tissue of the *AngII* group, reflective of inflammation that was also sustained in time in the *AngII*+*2W* and *AngII*+*3W* groups (Figure 2B). In line with the known proliferative effect of AngII in vascular tissue (36, 37), Ki67 labeling of aortic tissue was increased in the *AngII* group and, again, maintained in the *AngII*+*3W* group (Figure 2C).

### Oxidative Stress

AngII stimulates tissue nicotinamide adenine dinucleotide phosphate (NADPH) oxidases in cardiac and vascular cells to produce superoxide anions and, upon dismutation with extracellular SOD, the secondary oxidizing product H_2_O_2_ (38). Accordingly, plasma hydroperoxides were elevated in the *AngII* group, and also in the *AngII*+*2W* group (Supplementary Figure 1), reflecting persistent systemic oxidant stress at least up to 2 weeks after Ang II removal in our model.

### Comparative Transcriptomic Profiling of AngII vs. AngII “Memory” Conditions

To gain further mechanistic insight into the sustained effect of AngII on this vascular phenotype, we used an unbiased approach through whole aortic tissue RNA-sequencing. We first assessed the differential expression of genes and underlying signaling pathways in the *AngII* group compared with control, untreated mice. Next, we performed a similar comparison between the *AngII*+*2W* and *AngII*+*3W* groups (i.e., “memory” conditions) vs. control, untreated mice. We also compared the resulting list of genes and pathways to identify transcripts similarly modulated in *AngII* and “memory” groups, assuming that these sustained up and/or downregulated transcripts may be related to the observed “memory” phenotype.

**Figure 3:**
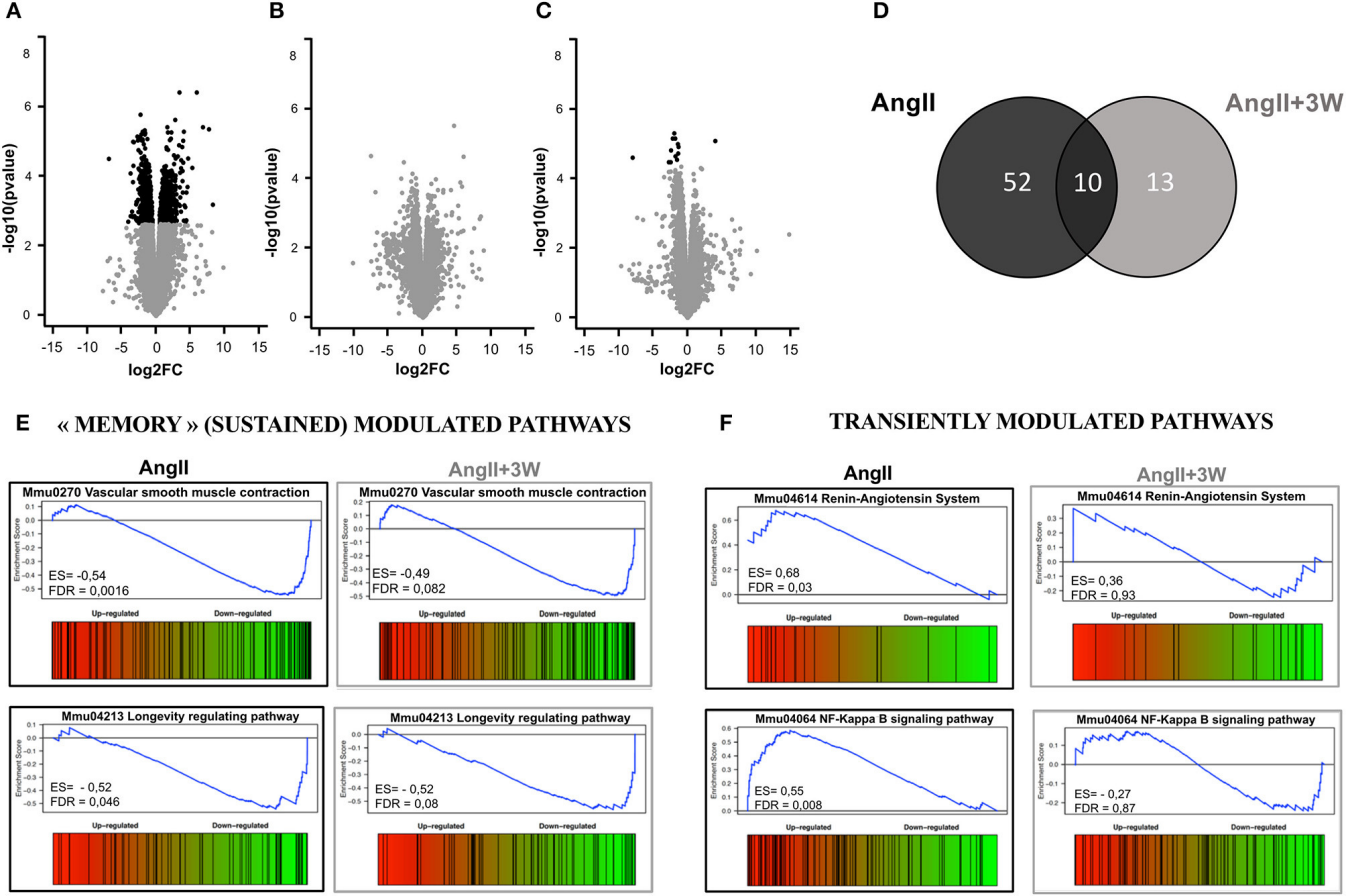
Transcriptomic profiling of aortic tissue from AngII memory model. Volcano plot representing the analysis of differential expression of genes in **(A)** *AngII* vs. *CTRL*; **(B)** *AngII*+*2W* vs. *CTRL* and **(C)** *AngII*+*3W* vs. *CTRL* data sets. Black points mark the genes with significantly (FDR < 0.05) increased or decreased expression compared to CTRL. The x-axis shows log_2_fold-changes in expression and the y-axis the –log_10_(*p*-value). **(D)** Gene set enrichment analysis (GSEA) from compiled KEGG pathways. Venn diagram showing the number of gene sets differentially modulated in each condition, and the overlap for 10 of them (FDR value < 0.1) **(E)** Enrichment score diagram illustrating the expression pattern of 2 of the 10 commonly regulated pathways (e.g., present in *AngII* and in *AngII*+*3w*, and modulated in the same direction) “vascular smooth muscle contraction” and “longevity regulating pathway”. **(F)** Enrichment score diagram illustrating the expression pattern of 2 transiently modulated pathways by Angiotensin II infusion: “Renin angiotensin System” and “NF KappaB signaling pathway”.

Volcano plots in Figures 3A–C represent differential expression of gene data sets comparing each condition to *CTRL*, considering FDR < 0.05 as a cutoff. As shown in Figures 3A,C, we found 808 genes significantly differentially expressed in *AngII* and 13 genes in *AngII*+*3W* memory condition. Unlike the *AngII* condition (Figure 3A) in which 55% of genes were upregulated, we found a vast majority of significantly underexpressed genes in *AngII*+*3W* memory condition (Figure 3C). As no significant differentially expressed gene was found with this FDR cutoff in the *AngII*+*2W* group (Figure 3B), we focused on the *AngII*+*3W* memory group compared with *AngII* for further analysis.

Gene set enrichment analysis of the 2 data sets is summarized in Figures 3D–F. A total of 62 KEGG pathways were significantly differentially modulated in *AngII* group, and 23 in *AngII*+*3W* group, using FDR < 0.1 as a cut-off. The Venn diagram in Figure 3D shows that 10 of them were commonly modulated pathways between the 2 groups. Among these 10 pathways, we next searched for those modulated in the same direction, e.g., up- or downregulated. Four of the 10 commonly modulated pathways were similarly downregulated, and identified as “dilated cardiomyopathy” (mmu05414); “hypertrophic cardiomyopathy” (mmu05410); “longevity regulating pathway” (mmu04213) and, notably, “vascular smooth muscle contraction” (mmu04270) (Figure 3E; Supplementary Figure 2). Conversely, other pathways known to be regulated by AngII, such as, “Renin angiotensin system” (mmu044614), “NF-KappaB signaling” (mmmu04064) and “cGMP signaling” (mmu04022) were, as expected, up (for the first two) or downregulated (for the latter) in the *AngII* group, but returned to normal level of enrichment in the *AngII*+*3W* “memory” group (Figure 3F; Supplementary Figure 2).

**Figure 4:**
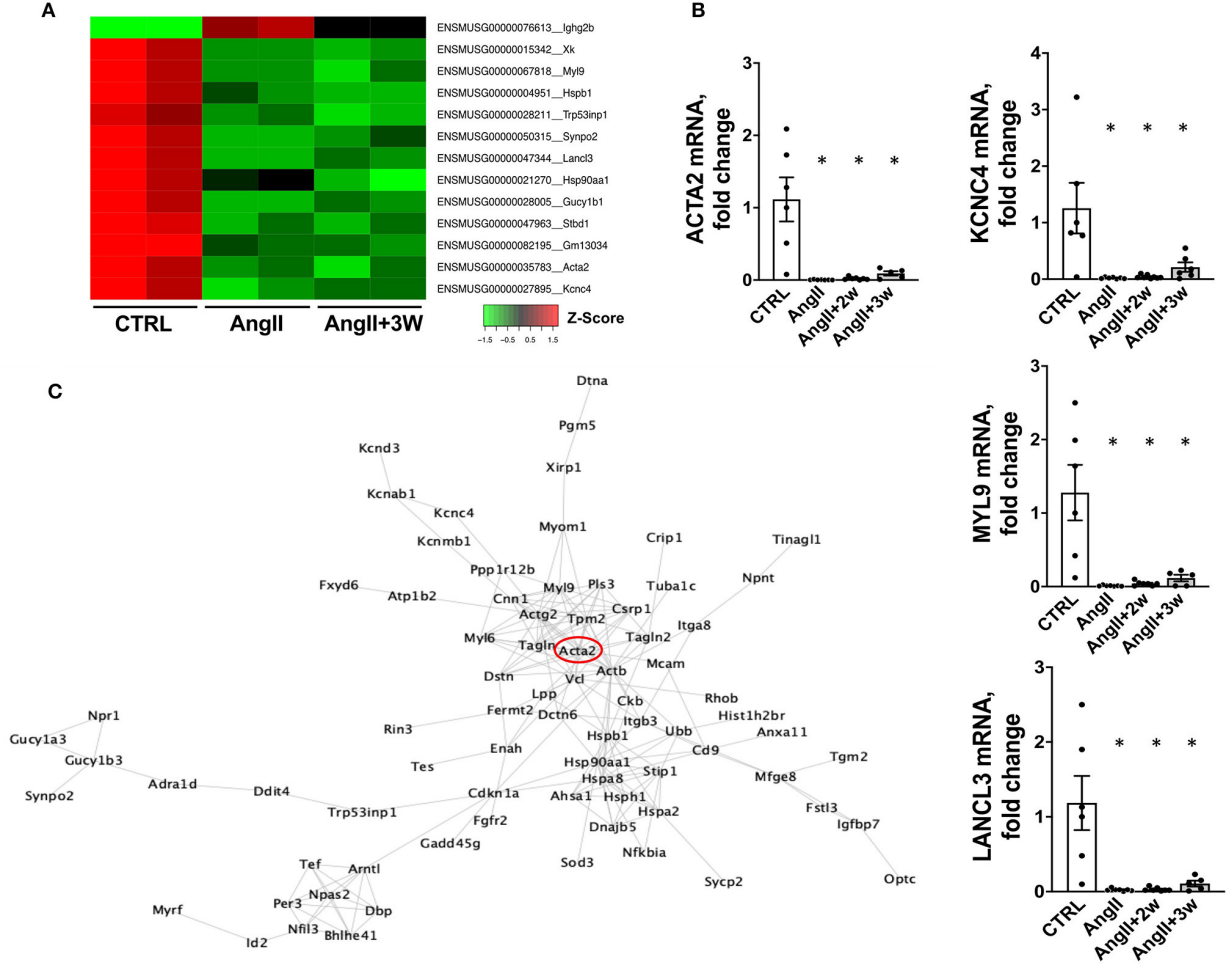
Common downregulation of specific genes in aortic tissue from AngII and “memory model” and corresponding interactome mapping. **(A)** HeatMap representing expression level (Z-score) of the 13 identified commonly modulated transcripts in AngII+3W and AngII vs. CTRL conditions. **(B)** RT qPCR on a replication group of mice confirming RNA seq expression data on selected genes: *Lancl3, Kcnc4, Myl9*, and *Acta2, n* = 25; *p < 0,05, one way ANOVA followed by Holm's-Sidak comparison test. **(C)** Protein–protein interaction network corresponding to differentially expressed genes in aorta in the “memory” condition (*AngII*+*3w*) vs. *CTRL*. To build a comprehensive picture, genes with FDR up to < 0,1 were included. Based on Cytoscape String Network database.

We next compared the gene lists to identify transcripts commonly regulated in both *AngII* and *AngII*+*3W* conditions. A total of 13 genes were commonly differentially expressed in both conditions, i.e., 13 genes similarly and significantly modulated both in the “memory” group vs. control, untreated mice and in the AngII group vs. control, untreated mice. Figure 4A represents a heat map with these 13 common transcripts, illustrating changes in the expression level in each condition (*AngII* and *AngII*+*3W*) compared with control, untreated condition (see also Supplementary Figure 3).

Among these, our attention was drawn to *Acta2*. *Acta2* is a gene coding for alpha-smooth muscle actin (αSMA), a protein of the cytoskeleton mainly expressed in smooth muscle cells that is involved in vascular contractility and blood pressure homeostasis. Mutations in this gene cause a variety of vascular diseases, such as thoracic dilated aortic disease, coronary artery disease, stroke, and Moyamoya disease (39), and also multisytemic smooth muscle dysfunction syndrome (40). Downregulation of αSMA expression induced by AngII has also been described in vascular SMC *in vitro* (41, 42).

To better delineate the putative role of *Acta2* in the context of our pathway enrichment analysis, we drew a virtual protein–protein interaction network using the Cytoscape software (fed from the String database). We only included the list of genes that were differentially expressed in *AngII*+*3W* memory condition (compared with *CTRL*), with enlarged FDR cut-off of < 0.1 for a more comprehensive picture (Figure 4C). Note that this interactome did not consider the direction of modulation (e.g., if a transcript was either up or downregulated, to the extent that protein abundance is modulated similarly). Interestingly, we observed that *Acta2* was located at a central hub of this protein–protein interaction network.

Based on the earlier observations, we focused on *Acta2* as a potential driver of our phenotype.

### Sustained Downregulation of Acta2 in a Replication Cohort: Putative Role of Myocardin and Histone Methylation

**Figure 5:**
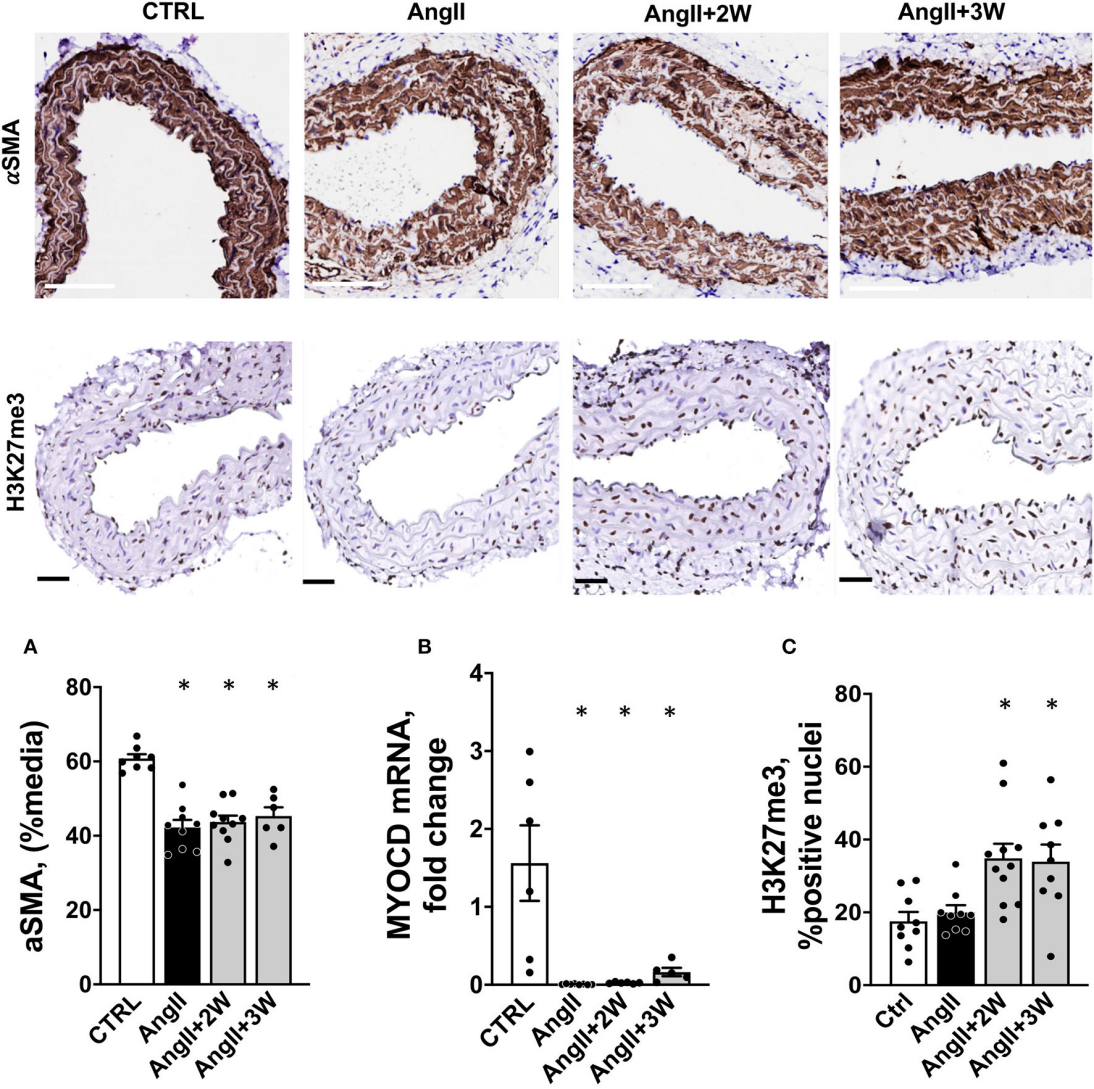
Coordinated regulation of Alpha-Smooth Muscle Actin, its transcription factor *Myocd* and histone trimethylation in aortic tissue from the Ang II “memory” model. **(A)** Sustained down regulation of αSMA protein in aortic tissue from a replication cohort by immunostaining (white bar = 100 μm). *n* = 33; * = *p* < 0.05, one way ANOVA followed by Dunett's multiple comparison test. **(B)** *Myocd* mRNA expression by RTqPCR in mouse aortas from a replication cohort, *n* = 26; *p < 0.05, one way ANOVA followed by Holm's-Sidak comparison test. **(C)** H3K27me3 staining and quantification in paraffin-embedded sections of aortas, expressed as % of positive nuclei (black bar = 50 μm), *n* = 39; *p < 0.05, one way ANOVA followed by Dunett's multiple comparison test.

We confirmed our RNAseq data in a replication cohort of identically treated mice, in which we found a downregulation of *Acta2* mRNA expression by RT-qPCR in *AngII*, and both *AngII*+ *2W* and *AngII*+*3W* groups, compared with *CTRL* (Figure 4B); among the 13 genes identified in the RNAseq data, downregulation of *Myl9, Kcnc4*, and *Lancl3* mRNA expression were also confirmed (Figure 4B). αSMA protein levels were also evaluated through quantitative immunostaining on aortic tissue. We confirmed a significant decrease in αSMA protein expression in the *AngII* group, which was significantly maintained in both “memory” conditions, *AngII*+*2W* and *AngII*+*3W* group (Figure 5A). Remarkably, the cytoskeleton in smooth muscle cells of the arterial media in these two groups was profoundly altered, with important structural defects and disorganization (see representative pictures in Figure 5).

Among factors controling αSMA expression are transcription factors Myocardin (*Myocd*) and Serum Response Factor (*SRF*). Altogether they form a complex which binds CArG (CCA/T_(rich)_GG) sequence motif upstream *Acta2* (and other contractile genes), *SRF* serving as doking platform for *Myocd* activity, leading to active contractile transcription machinery. In our RNAseq data, we observed that *Myocd* and *SRF* transcripts were significantly downregulated under AngII (−2,07 log_2_FC, FDR = 0,02), with the same trend in the memory condition for *Myocd* (−1,42 log_2_FC, FDR= 0,13). We decided to evaluate *Myocd* expression by RT qPCR in our replication group of mice. We observed that this transcription factor was robustly repressed under AngII infusion and that this repression was sustained in time despite the end of the pharmacological stimulation (Figure 5B).

To gain further understanding of the sustained downregulation of *Acta2* and *Myocd*, we examined putative epigenetic regulatory mechanisms. Indeed, our pathway analysis using ORA or GSEA, identified significant enrichement of several epigenetic pathways under AngII such as “HDAC's deacetylase histone” (R-MMU-3214815), “HATs acetylate histone” (R-MMU-3214847), and also “PRC2 methylates histone and DNA”(R-MMU-21230) (see Table I in Supplemental Material, Reactome T1 ORA, Reactome T1 GSEA). Interstingly, PRC2 is a protein complex that keeps transcriptionally silent genes in a repressed state by trimethylating histone H3 on lysine 27. We then evaluated the status of this epigenetic mark in our model, using immunostaining on aortic sections. Notably, this revealed a significant increase in H3K27me3, only in the 2 memory conditions, *AngII*+*2W* and AngII+3W (Figure 5C).

### *In vitro* AngII Memory Model in Human Aortic Vascular Smooth Muscle Cells

**Figure 6:**
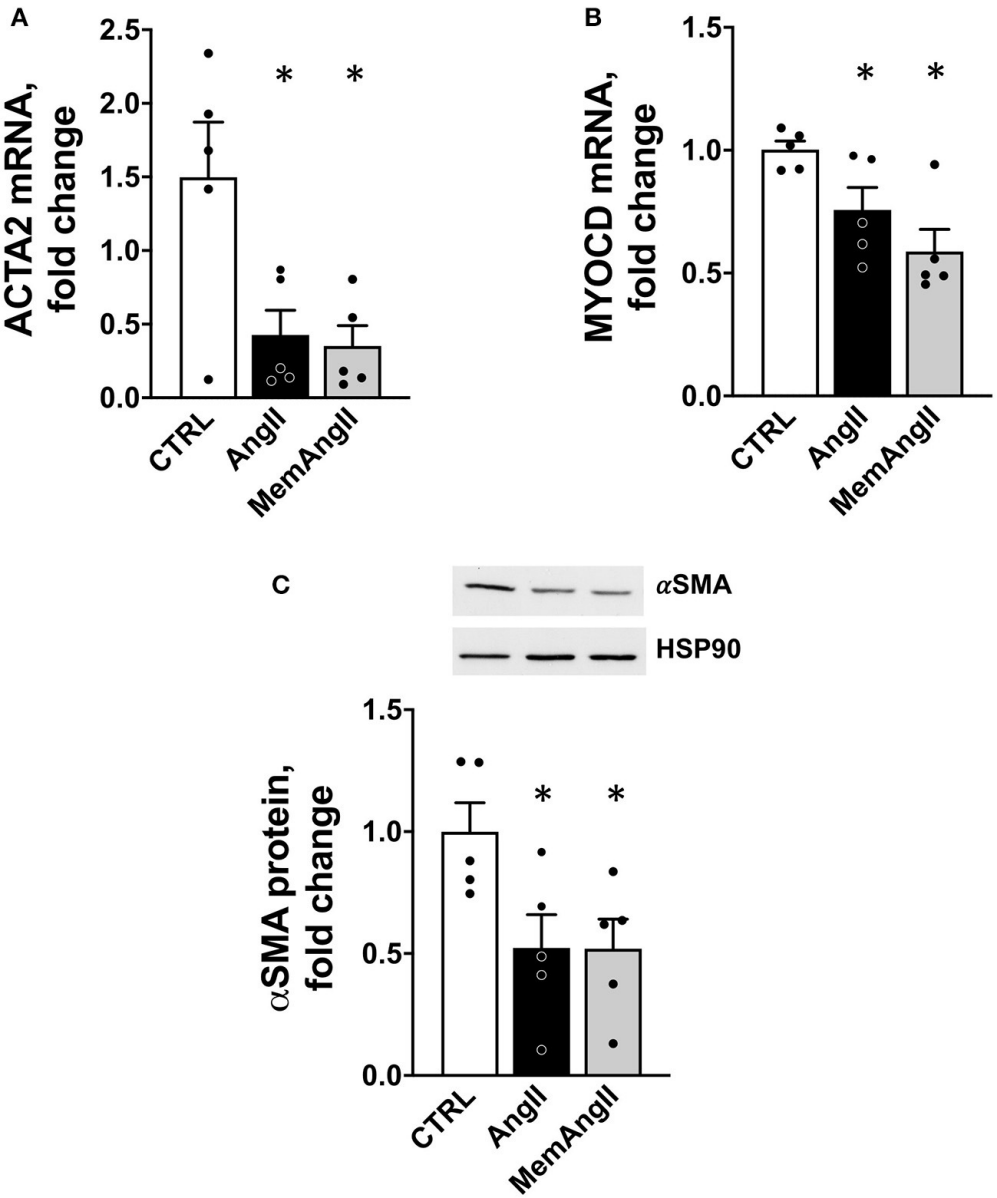
Downregulation of *ACTA2* and *MYOCD* in a homotypic cell culture model of human vascular smooth muscle cells reproducing the AngII “memory” effect *in vitro*. **(A)** *ACTA2* mRNA expression (by RT qPCR) in HAVSMC exposed continuously (*AngII*) or transiently (*MemAngII*) to AngII compared with the control cells maintained in serum-deprived control media. **(B)** *MYOCD* mRNA expression in the same *in vitro* model **(C)** aSMA protein level in the same model, *N* = 5 independent experiments, *p < 0.05 one way ANOVA followed by Sidak's multiple comparisons test.

To verify these observations in a homotypic cell system of human origin, we developed an *in vitro* model of AngII memory on cultured human aortic VSMCs. Cells were exposed to continuous AngII at 1μM for 72 h (*AngII*); or to the same serum-deprived control medium for 72 h (*CTRL*), or 48 h of AngII, followed by 24 h of control medium (*MemAngII*). First, we observed the same downregulation of *ACTA2* under AngII stimulation, as depicted by mRNA expression and protein levels (Figures 6A,C). Notably, this downregulation was also maintained in the “memory” condition. This was paralleled with a downregulation of mRNA expression of the transcription factor *MYOCD*, upon continuous AngII, as well as in the “memory” condition (Figure 6B).

## Discussion

The main findings of this study can be summarized as follows: (i) an initial 2-week exposure to AngII induces profound changes in cardiac and vascular remodeling, including endothelial activation, vascular inflammation and oxidant stress, all of which are maintained up to 3 weeks after AngII withdrawal; notably, this phenotype is sustained despite early normalization of blood pressure after AngII withdrawal, a proxy to a “legacy” or “memory” effect in this mouse model; (ii) comparison of the transcriptomic profiles at the end of the 2-week Ang II treatment (*AngII* group) or 3 weeks after Ang II withdrawal (*AngII*+*3 weeks* group) identified 13 commonly regulated transcripts (1 up and 12 down), and a set of 4 commonly modulated pathways by GSEA, some of which point to altered structural or contractile properties of the arterial wall. Conversely, many other transcripts classically associated with AngII effects and (mostly) upregulated in the *AngII* group, are not persistently regulated in the *AngII*+*3 weeks* group; this highlights the above 13 genes, corresponding to the sustained downregulated transcripts, as potential targets for a “memory” effect; (iii) among these, *Acta2* is a likely candidate, as first confirmed in a replication cohort, including at the protein level, but also from our observation of striking downregulation of Myocardin (*Myocd*), its transcriptional coregulator and from changes in histone methylation, as corresponding epigenetic repressive marks, in aortas of both the memory groups, *AngII*+*2W* and *AngII*+*3W* groups.

Ours is one of the few studies examining the effect of AngII on the full transcriptomic profile of mouse aortic or arterial tissues in “wild-type” (C57Bl6/J) mice. In a microarray study on ApoE-/- mice-treated with AngII, Rush et al. identified genes overexpressed in mice that did not develop aneurysms under pharmacological Ang II stimulation, i.e., protective against abdominal aortic aneurysms (20). Consistent with their results, a majority of those transcripts were significantly downregulated in our *AngII* group of mice, such as *Sost, Dstn*, or *Hspaa1a* (see Supplementary Data). Our profiles are also in line with results by Spin et al. identifying genes decreased in prematurely ruptured anevrysm in AngII-treated ApoE-/- mice (e.g., *Bmp6, Ltbp1, Rock1*) (43). Another transcriptomic analysis of 3 differential arterial beds of C57Bl6 mice treated with AngII identified *Sphk1* as commonly modulated transcript in the 3 types of tissue. This transcript is also upregulated under AngII in our RNAseq data, albeit not significant with an FDR cutoff of 0.05. Other transcripts such as *Thy1* or *Htatip2*, described to be overexpressed in thoracic aorta, are similarly regulated in our *AngII* group. Notably, as we did, the same study identified a downregulation of *Gucy1b3* transcripts (corresponding to the beta subunit of soluble guanylyl cyclase) in thoracic and abdominal aortic tissue, expected to be associated with a reduced vascular relaxation (21). Finally, in a recent study in C57Bl6 mice treated for a longer time with lower dose of AngII, Lv et al. identified 773 genes differentially expressed in aortas of hypertensive mice (22). As in this study, under AngII, mostly were upregulated genes (22). Among identified transcripts, we confirmed the overexpression of IGF1 in our RNAseq data in AngII-infused mice.

Even fewer studies have attempted to develop an AngII “memory” model (16, 17, 44) and in none of them was a transcriptomic profiling performed. In concordance with the only 2 studies in mice found in the literature, we observed sustained macroscopic and microscopic cardiac hypertrophy (16); ICAM-1 endothelial expression, inflammation in the vascular wall (e.g., CD45-positive cell infiltration), and oxidative stress (17) that persisted after AngII withdrawal. A major difference in the aforementionned study compared with ours is that blood pressure remained elevated 1 week after the end of AngII infusion, the only “remote” time-point examined (17). This could simply be explained by the different AngII dosage and timing for the “memory” condition (1 week vs. 2 weeks after the end of AngII infusion). Although the distinction between differential delays for phenotype reversal (e.g., slower for tissue remodeling, more rapid for blood pressure) vs. “memory” effect could be argued, the present model shows the persistence of remote effects (up to 3 weeks) despite clear evidence of termination of the initial stimulus (normalization of blood pressure). At the very least, and contrary to the previous study, it excludes that the long-lasting effects on remodeling result from sustained high blood pressure.

Another strengh of this study is the use of an unbiased approach to understand our phenotype through transcriptomic profiling. While differential transcript regulation did not reach the more stringent significance level in the *AngII*+*2W* group probably because of insufficient statistical power, the genes differentially expressed in the *AngII*+*3W* group at FDR < 0.05 were similary regulated in *AngII*+*2W* (Supplementary Data), suggesting a continuum in their regulation.

AngII, among other factors, promotes a switch in vascular smooth muscle cells from a contractile to “secretrory” phenotype (45). Through AT_1_R, AngII stimulation produces changes in the contractile machinery, constisting in decreased expression of contractiles markers such as αSMA (*ACTA2*), SM22a (*TAGLN*), and SMMHC (*MYH11*) (9, 46). As others (41, 47), in parallel with vascular remodeling, we observed significant changes in contractile markers expression in aortas under AngII infusion, with downregulation of “vascular smooth muscle contraction” pathway, and *ACTA2* expression, but also *TAGLN* and *MYH11* (see raw data). In addition, this phenotypic switch was maintained in time despite the end of AngII stimulation, for the “vascular smooth muscle contraction pathway”, and also for αSMA expression, both at mRNA and protein levels (Figures 4B, 5A). Among factors controling VSMC phenotypic plasticity are transcription factors Myocardin (*Myocd*) and Serum Response Factor (*SRF*). Altogether they form a complex which binds CArG (CCA/T_(rich)_GG) sequence motif upstream contractile genes, with SRF serving as doking platform for *Myocd* activity, leading to active contractile transcription machinery. Conversely, Krupple-like Factor 4 (*KLF4*) and ETS domain containign protein-1 (*ELK1*) binding to G/C repressor element, inhibit *Myocd/SRF* complex, leading to decressed expression of SMC differentiation markers (46). In our RNAseq data, we observed that *Myocd* and *SRF* transcripts were significantly downregulated under AngII (- 2,07 log_2_FC, FDR = 0,02), with the same trend in the *AngII*+*3W* memory condition for (-1,42 log_2_FC, FDR = 0,13). This was not the case for KLF4 and ELK1 (see raw data). We confirmed this downregulation of *Myocd* transcripts by RT-qPCR, including in the 2 memory conditions (*AngII*+*2W* and *AngII*+*3W* group) (Figure 5B). Interestingly, we could reproduce same results in HAVSMC in culture, in which we observed a sustained down expression of *ACTA2* and protein αSMA induced by AngII, associated with reduced expression of transcription factor *Myocd*; and despite removal of the pharmacologic stimulus. This makes *MYOCD* a likely candidate for upstream, sustained downregulation of *ACTA2* expression in AngII “memory” context.

Epigenetic regulation may also account for the sustained alteration of the vascular phenotype in the “memory” groups *in vivo*. Indeed, Histones H3 and H4 associated with CArG-containing regulatory elements of SM-MHCs (*MYH11*), SM22a (*TAGLN*), and αSMA (*ACTA2*) have been shown to be acetylated (a characteristic of chromatin accessibility) in contractile SMCs, facilitating SRF binding to the CArG box (48). Increased histone acetyltransferase (HAT) activity stimulates SM22a expression whereas increased histone deacetylases (HDACs) prevent SM22a expression (49). Consistently, our pathway analysis using ORA or GSEA, identified significant enrichement of several epigenetic pathways under AngII (such as “HDACs deacetylase histone” (R-MMU-3214815), “HATs acetylate histone” (R-MMU-3214847).

Other modalities of epigenetic regulation may also be at play. H3 histone dimethylation in lysine 4 (H3K4me2) is a marker of differentiated SMCs and is maintained even if SMCs undergo phenotypic modulation (50). DNA demethylation by TET2 increases DNA accessibility to transcription factors resulting in increased SMC differentiation marker expression (51). Of interest, the *ACTA2* promoter was shown to be hypermethylated (a usual mark of repressed expression) in genome-wide methylation studies in human atherosclerotic aortas (52, 53). As we observed sustained downregulation of *Acta2* transcripts in our “memory” condition, an AngII-induced methylation leading to repressive imprinting and phenotypic switch might well be involved. Again, our RNAseq pathway analysis suggests the involvement of epigenetic regulators involved in methylation, such as “PRC2 methylates histone and DNA”(R-MMU-21230) (see Supplementary Data, Reactome T1 ORA, Reactome T1 GSEA). In particular, PCR2 complex catalyzes trimethylation of histone H3 on lysine 27 (H3K27me3), a histone mark necessary for maintaining transcriptional repression during multicellular development. Cell type-specific patterns of H3K27me3 are crucial for preserving cell identity (54). Consistent with this analysis, we observed a significant increase in H3K27me3 epigenetic mark in the aortic tissue and, intriguingly, only in both memory conditions (Figure 5C). However, the signaling elements involved in this response to AngII remain to be studied in more details.

## Conclusion

Altogether, our observations support a “memory” effect sustained beyond AngII-induced hypertension and leading to downregulation of specific gene expression, such as *Acta2*, and vascular injury. Future characterization of the underlying AngII-dependent signaling might unveil new targets for its therapeutic modulation and reversal of this adverse legacy effect.

## Data Availability Statement

The datasets presented in this study can be found in online repositories. The names of the repository/repositories and accession number(s) can be found below: https://www.ncbi.nlm.nih.gov/geo/query/acc.cgi?acc=GSE175588.

## Ethics Statement

The animal study was reviewed and approved by Institutional Animal Care and Research Advisory Committee of the Université Catholique de Louvain.

## Author Contributions

LP, CD, and J-LB designed project and experiments. LP wrote manuscript and designed figures under J-LB supervision. J-LB reviewed and corrected, and other authors reviewed and commented. CB performed immunohistochemistry experiments and analysis. LP, BB, and JA performed RNAseq anaylsis (LP: RNA extraction and interactome, BB: pre-analytic and libraries, JA: bioinformatic analysis). LP, RV, DD, HE, CF, and LM performed in *in vivo* and *in vitro* experiments. HE especially for telemetries. All authors contributed to the article and approved the submitted version.

## Funding

This study was funded by Belgian Fond National de la Recherche Scientifique (FNRS, CDR J.309.21) and Fondation Saint Luc (Grant Pierre de Merre).

## Conflict of Interest

The authors declare that the research was conducted in the absence of any commercial or financial relationships that could be construed as a potential conflict of interest.

## Publisher's Note

All claims expressed in this article are solely those of the authors and do not necessarily represent those of their affiliated organizations, or those of the publisher, the editors and the reviewers. Any product that may be evaluated in this article, or claim that may be made by its manufacturer, is not guaranteed or endorsed by the publisher.
