# Supplementary material for: Sustained Downregulation of Vascular Smooth Muscle Acta2 After Transient Angiotensin II Infusion: A New Model of “Vascular Memory”
Source: Front Cardiovasc Med. 2022 Mar 14;9:854361. doi: 10.3389/fcvm.2022.854361 (PMC8964264; doi:10.3389/fcvm.2022.854361)
Supplement: Supplementary file 1 [file Data_Sheet_1.doc]

**Supplemental materials “Sustained down-regulation of vascular alpha-smooth muscle actin after transient Angiotensin II infusion: a new model of “vascular memory”.**

1. **Expanded material and methods**
2. ***Implanted telemetries***

Implants (DSI, USA) were surgically inserted as following: under anaesthesia with a mixture of ketamine and xylazine, the left common carotid artery was isolated and the tip of the catheter was retrogradely inserted into the aorta until the aortic arch. The catheter was connected to the body of the implant, placed in a subcutaneous pouch in the right flank. After 5 days of recovery, short- or long-term (24 hours) online recordings were digitized (range, 20 to 2000 Hz) and stored for further analysis. Recordings were also obtained during the resting period of the animals, under visual monitoring of their activity, for maximal reproducibility.

1. ***Plasmatic peroxides***

Systemic oxidative status was evaluated by measurement of hydroperoxides plasmatic levels using a d-ROM kit based on Fenton’s reaction (Diacron International, MC001). Briefly, in living animals under anesthesia with ketamine, blood was collected by retro-orbital puncture through heparinized glass pipet, centrifuged for red blood cells separation, and plasma was immediately frozen in liquid nitrogen. Thawed plasma was used later according manufacturer’s recommendations. The formation of reactive products was registered at 505 nm by SpectraMax i3 microplate automatic photometer (Molecular Devices, LLS, USA).

1. ***List of primers***

|  | **Fw** | **Rv** |
| --- | --- | --- |
| Mm-ACTA2 | ACTACTGCCGAGCGTGAGAT | CTTCTCCAGGGAGGAAGAGG |
| Mm-MYL9 | TGTGGAGTTGTCTCAGCACC | CAGCCTTGTATTAAGAGACTGTCC |
| Mm-LANCL3 | GAAACCGCAGTACCTGGACA | TGGCATCACGCTGTCTTCAT |
| Mm-KCNC4 | GGGACAGCACCTACAGTGAC | TCCCTCCTCATCAGACAGCA |
| Mm-GAPDH | TGCACCACCACCTGCTTAGC | GGATGCAGGGATGATGTTCT |
| Hs-ACTA2 | ACTGAGCGTGGCTATTCCTCCGTT | GCAGTGGCCATCTCATTTTCA |
| Hs-GAPDH | GGAGTCAACGGATTTGGTCGTA | GGCAACAATATCCACTTTAACAGAGT |
| Mm-MYOCD | CTGTGTGGAGTCCTCAGGTCAAACC | GATGTGCTGCGGGCTCTTCAG |
| Hs-MYOCD | AGGTGGGGCCAAAGTTTTCA | AGGACAGCAGTTGGACTTCG |

1. **Supplemental figures**

**Supplemental figure 1. Oxidative stress measurement in AngII memory model in vivo**. **Quantification of plasma peroxides**, expressed as fold change of ctrl mice *p< 0.05; One-way ANOVA followed by Dunett’s multiple comparisons test, n=40.


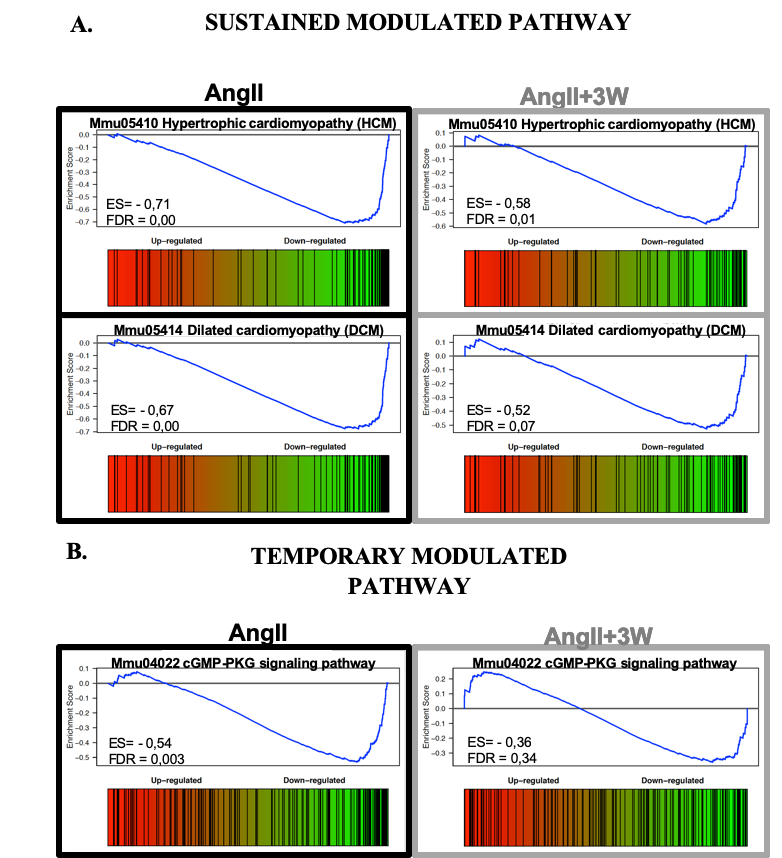


**TRANSIENTLY MODULATED PATHWAYS**

**Supplemental figure 2.** **Gene set enrichment analysis (GSEA) from compiled Kegg pathways. A. Enrichment score diagram** of 2 supplemental commonly modulated pathway in *AngII* and *AngII+3W* groups: “dilated cardiomyopathy” and “hypertrophic cardiomyopathy”. **B**. **Enrichment score diagram** illustrating “**cGMP-PKG signaling pathway**” downregulation under AngII that was not sustained after AngII interruption.

|  | **AngII** | | **AngII+3W** | | **AngII+2W** | |
| --- | --- | --- | --- | --- | --- | --- |
| **gene_name** | **Log_2_FC** | **FDR** | **Log_2_FC** | **FDR** | **Log_2_FC** | **FDR** |
| ***Ighg2b*** | 5.94 | 0.003 | 4.03 | 0.036 | 4.53 | 0.053 |
| ***Xk*** | -1.95 | 0.006 | -1.95 | 0.036 | -1.04 | 0.173 |
| ***Myl9*** | -2.09 | 0.006 | -2.17 | 0.036 | -1.03 | 0.215 |
| ***Hspb1*** | -1.44 | 0.012 | -1.81 | 0.036 | -0.986 | 0.173 |
| ***Trp53inp1*** | -1.1 | 0.014 | -1.42 | 0.036 | -0.244 | 0.578 |
| ***Synpo2*** | -2.15 | 0.006 | -1.77 | 0.043 | -0.944 | 0.244 |
| ***Lancl3*** | -2.64 | 0.006 | -2.42 | 0.039 | -1.25 | 0.215 |
| ***Hsp90aa1*** | -0.716 | 0.030 | -1.33 | 0.036 | -0.743 | 0.202 |
| ***Gucy1b1*** | -1.62 | 0.006 | -1.38 | 0.041 | -0.921 | 0.173 |
| ***Stbd1*** | -1.49 | 0.012 | -1.55 | 0.045 | -0.904 | 0.215 |
| ***Gm13034*** | -6.87 | 0.011 | -8.01 | 0.044 | -7.58 | 0.137 |
| ***Acta2*** | -2.18 | 0.013 | -2.47 | 0.045 | -1.35 | 0.244 |
| ***Kcnc4*** | -3.34 | 0.0062 | -2.74 | 0.045 | -1.68 | 0.202 |

**Supplemental figure 3**. **Common downregulation of specific genes in aortic tissue from AngII memory model.** Differential expression level (log_2_FC) of the 13 identified commonly modulated transcripts in AngII+3W and AngII versus CTRL conditions, with FDR <0.05. Differential expression level (log2FC) of the same 13 transcripts and respective FDR values in the other memory condition (AngII+2W).
